# AIoT Used for COVID-19 Pandemic Prevention and Control

**DOI:** 10.1155/2021/3257035

**Published:** 2021-10-13

**Authors:** Shu-Wen Chen, Xiao-Wei Gu, Jia-Ji Wang, Hui-Sheng Zhu

**Affiliations:** ^1^School of Math and Information Technology, Jiangsu Second Normal University, Nanjing 211200, China; ^2^State Key Laboratory of Millimeter Waves, Southeast University, Nanjing 210096, China

## Abstract

The pandemic of COVID-19 is continuing to wreak havoc in 2021, with at least 170 million victims around the world. Healthcare systems are overwhelmed by the large-scale virus infection. Luckily, Internet of Things (IoT) is one of the most effective paradigms in the intelligent world, in which the technology of artificial intelligence (AI), like cloud computing and big data analysis, is playing a vital role in preventing the spread of the pandemic of COVID-19. AI and 5G technologies are advancing by leaps and bounds, further strengthening the intelligence and connectivity of IoT applications, and conventional IoT has been gradually upgraded to be more powerful AI + IoT (AIoT). For example, in terms of remote screening and diagnosis of COVID-19 patients, AI technology based on machine learning and deep learning has recently upgraded medical equipment significantly and has reshaped the workflow with minimal contact with patients, so medical specialists can make clinical decisions more efficiently, providing the best protection not only to patients but also to specialists themselves. This paper reviews the latest progress made in combating COVID-19 with both IoT and AI and also provides comprehensive details on how to combat the pandemic of COVID-19 as well as the technologies that may be applied in the future.

## 1. Introduction

Coronavirus disease 2019 was officially named “COVID-19” by the WHO in February 2021. Since the first confirmed case of COVID-19, researchers in more than 30 countries and regions around the world have been actively searching for the ways to control and treat COVID-19. As the most updated and popular technology in the 21^st^ century, the system of IoT can realize data informatization, remote control, and intelligent management and monitoring through real-time network. Therefore, it is of great significance to apply AI-assisted IoT technology (Alito) to clinical medicine, especially in this context of COVID-19 pandemic prevention and control work.

Alito system control of COVID-19 is important not only for patients but also for the general public. People can use wearable devices to independently monitor, observe, and record their respiration rate, heart rate, daily body temperature, and other physiological values, so as to make judgment on their own. Even in the state of being isolated, they can also quickly get to know changes in their vital signs. More remarkably, the application of AIoT in the front line of clinical practice can boost the development of modern intelligent medical care, such as remote screening, intelligent diagnosis, and remote intensive care, so it is perceived as a great breakthrough for conventional pandemic prevention and control. Remote screening makes it unnecessary to screen people in emergency rooms in large numbers, thereby reducing the possibility of being exposed to viruses. Intelligent diagnosis can help to solve the problem of slow speed and low accuracy of manual reading of image scanning reports. As an auxiliary method, intelligent diagnosis can help front-line doctors to quickly determine whether patients are infected with COVID-19, isolate patients in the first place, and take steps of treatment. Remote intensive care is applied to patients who have already been infected with COVID-19. Even after these cured patients leave the hospital, doctors can utilize 5G technology, Wi-Fi, or other third-party mobile devices to get to know the changes in their vital body signs and then offer them due suggestions and advice, which can be seen as one part of telemedical development. Furthermore, under the circumstances of the shortage of medical staff or facility, telemedicine can help to solve this problem to a certain degree, without human mutual contact and possibility of cross-infection. AIoT not only plays a major role in clinical medical treatment but also facilitates the management of the public society. From the basic agricultural/industrial chain to the construction of intelligent infrastructure, AIoT can help the whole human society to resist novel coronavirus with its own characteristics. With the pandemic being gradually brought under control, more and more enterprises, whether large or small, are applying AIoT technology to resume their work and production, trying to get rid of the dilemma brought about by the pandemic. On the other hand, more and more countries and regions are applying 5G and robots to fight against COVID-19 pneumonia.

Section 2 introduces the prevention and control methods of the pandemic: wearable devices with Wi-Fi, 5G, and Bluetooth as the main technologies.  Section 3 introduces the methods of remote screening.  Section 4 discusses the methods of intelligent diagnosis.  Section 5 demonstrates some examples of remote intensive care.  Section 6 describes the development and management of public society with the help of AIoT technology.  Section 7 introduces the application of 5G and robotics technology in the fight against the pneumonia.  Section 8 gives a summary and prospect of AIoT technology. With the help of AI, these new IoT technologies will drive the development of modern pandemic prevention and control.

This review mainly discusses the advantages and disadvantages of different methods in remote screening, intelligent diagnosis, and remote intensive care, based on the COVID-19-related studies before May 31, 2021. It also studies how the public society and enterprises of all sizes can use AIoT for recovery and development in the context of the pandemic. It is hoped that it can provide some help and guidance for doctors and researchers to overcome COVID-19.

## 2. New Methods for COVID-19 Prevention and Control: Wearable Devices

**Figure 1:**
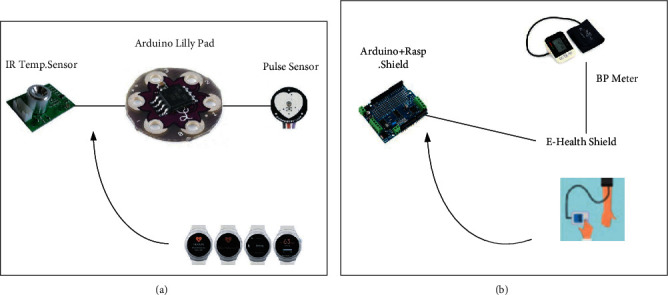
Wearable device and nonwearable device module: (a) wearable gadgets; (b) nonwearable gadgets.

Wearable technology, first developed at a MIT lab in the 1960s, embeds such technologies as multimedia, sensors, and wireless communications into people's clothing. As novel coronavirus is wreaking havoc worldwide, wearable devices based on AIoT systems are used to measure COVID-19-related signs, such as respiratory rate and body temperature. Mohammed et al. [1] propose the use of a smart helmet with a mounted thermal imaging system to automatically detect coronavirus through the thermal imaging system, thereby reducing the human mutual contact. A positioning system is located in smart helmets. The system will automatically respond when detecting higher-than-normal temperatures. The location system module immediately marks and determines the geographic location, while sending notices to the designated smartphone via GSM. That way, healthcare workers can obtain timely data on people's body temperature. However, as the second-generation mobile communication system, the Global System for Mobile Communications (GSM) lags behind the new era of 5G technology. In China, 5G technology is slowly maturing. For example, Guangqi Technology introduces a 5G-based smart helmet at the new China International Import Expo. The data show that the smart helmet adopts advanced low-power design, while the system saves 85% of power consumption. Meanwhile, the standby time of the whole machine is more than 72 hours, and the identification function can last for 6 hours continuously. It is predicted that after the smart helmet is put into use, the hazard can be detected on two of every 10,000 people identified and can be dealt with straightaway. Still, how can the performance of smart helmets be improved for those countries with underdeveloped 5G technology? The suggestion is to combine the GSM module with special mobile applications. The information is sent through the GSM module, and all details are viewed in the mobile applications. Only in this way can users be effectively monitored. Fyntanidou's team [2] has developed a wrist-worn wearable device that uses advanced digital signal processing algorithms to continuously extract heart rate, oxygen saturation, body temperature estimates, etc. Like smart helmets, wrist-worn wearable devices can transmit processed data to an app or a designated third-party mobile port in a timely manner through mobile communication protocols. In particular, they have developed an application specifically for emergency patients, which can be connected to the patients' wrist-worn wearable device to timely observe the changes in the patients' vital signs, which can serve as auxiliary information to help doctors to make medical judgments on the patients. However, on the other hand, the shortcomings of this device have limited its development, including the use of volatile memory, the working ability of battery, and other factors. The study in [3] proposes a smart limbic system, as shown in Figure 1, which relies on wearable devices to detect those at risk of infection. The system uses wearable modules equipped with infrared and pulse sensors to calculate body temperature and pulse rate in real time. The other unportable module is placed in crowded places, such as airports and shopping malls. This module is used to monitor respiratory and blood pressure data of suspected patients. The two modules operate and alert each other when a suspected case occurs in any public place. However, it is hard to guarantee that the nonwearable modules are installed in the public places where suspected patients have visited. Meanwhile, the placement of modules also brings some security risks. The study in [4] uses a soft wearable sensor device attached to the body or placed in the sternal incision to receive respiration, heart rate, and other data, which can help to find the physiological changes caused by respiratory diseases. In the paper, because the given results are obtained in the laboratory, the relevant conclusions may be too idealized. It is hoped that novel coronavirus pneumonia can be monitored by other researchers in the future. Wearable sensors provide a novel way to detect COVID-19, which depends on recording changes in heart rate, respiration, cough, and body temperature over a long period of time to determine the coronavirus infection. These technologies, as well as data networks, mainly rely on Wi-Fi, 5G, and Bluetooth technology. However, only relying on such technology for continuous monitoring may lead to low accuracy and data loss problems.

**Figure 2:**
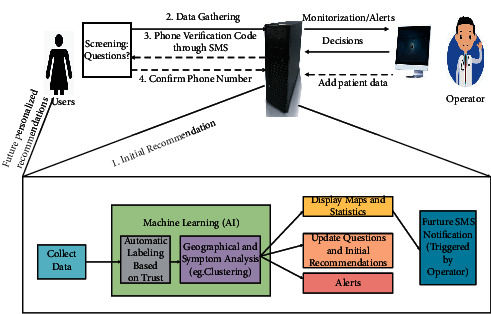
Architecture of the platform.

Wearable devices are widely used for COVID-19 control to measure the health status of potentially infected people and self-detect physiological changes during isolation. Wearable devices use GPS data to track the location, so that doctors can easily track a patient's condition. For example, the smart helmet proposed by the study in [1] has a mounted thermal imaging system to automatically detect coronavirus from thermal images without human intervention. This saves time and reduces human interactions that can cause the coronavirus to spread more quickly. Wearable devices such as smart watches can also be used to collect self-reported symptom-tracking data to distinguish between negative and positive cases among infected people. Head-wearable devices for detecting and tracking symptoms of COVID-19 can help to detect respiratory and heart rate problems by using such simple devices as headphones and mobile phones. Wrist-wearable devices can be used specifically for emergencies, such as prioritizing and classifying emergency patients. The study in [5] designs a wearable device for COVID-19 patients to predict the severity of the viruses. By developing an algorithm that predicts the progress of a virus from one stage to another, the device monitors information about the patient's health and alerts doctors when it predicts that the virus will move to the next stage. Using this method can prevent the deterioration of the patient's condition. In particular, the device is very useful for those patients who are in the state of self-isolation and who are not timely informed of their condition, even if they do not have professional medical knowledge. In addition, the 3D wearable prototype design proposed by Bassam et al. [6] includes wearable body sensors, network application programming interface layer, and mobile frontend layer. It is also the application of a portable wearable device to build an automated healthcare system. One of the advantages of the healthcare system is that COVID-19 can be detected at an early stage.  Figure 2 is the architecture of the system. The wearable sensor layer is used to measure temperature, heartbeat, oxygen saturation, and cough technology. Compared with the intelligent limbic system of [3], this system pushes the GPS location data of patients to medical units in real time, so as to more conveniently and effectively monitor patients and suspected patients. It is extremely suitable for emergency treatment. When the patient violates the self-isolation rules, the device will immediately send an alarm and notification signal. In the future, the AIoT-based technology of wearable devices will provide various methods to identify, perceive, and monitor coronavirus, which boosts great potential and prospect in the healthcare system. All the above-mentioned wearable devices are summarized in Table 1.

## 3. Remote Screening Based on AIoT

In the context of the COVID-19 pandemic, timely screening infected people as soon as possible is an important measure to prevent and control the pandemic. Indeed, manual screening is very slow, while remote screening technology can improve the speed and efficiency of screening. Therefore, remote screening based on the IoT system must be developed. Schinköthe's team [7] has built a free AIoT-based caregiver cockpit (C19CC). One user is first connected to a healthcare worker, who quickly categorizes the user according to the user's description. Color codes represent the severity of the examinees. For example, red codes represent COVID-19 patients, so examinees with red codes are automatically screened out, with their personal information and recent range of activity being marked out. C19CC can not only be used in remote screening but also be widely used in remote monitoring, hospital wards, etc. C19CC prescreens patients in a contact-free manner and quickly learns about those who urgently need treatment. In addition to conventional remote screening methods, lung ultrasound imaging classifiers are used for screening or diagnosis of COVID-19. Tan and Liu [8] propose a basic method based on facial recognition. The main function of this method is to remotely screen out suspicious patients through thermal images and then retrieve people who have close contact with them through a facial recognition system, so as to isolate people in time and prevent the spread of the virus. Although facial recognition can be used for initial remote screening, this method is extremely inefficient compared to other methods [9]. The portable scanner proposed by Hou et al. [10] will input the collected lung ultrasonic images to the platform. After the data are processed by the platform, it will classify the data in the subspace network. This sequence of steps allows the screening of patients with COVID-19. The classifier can be used on a large scale in nursing homes with adequate budgets, completely avoiding the possibility of infection among elderly people who hardly go to hospital. Because a COVID-19 patient often shows the fever symptom, the facial recognition system will also be used to detect the patient and alert the hospital platform via the Internet or mobile devices, so that the patient can be isolated and further diagnosed. The study in [11] proposes a method to analyze throat images and experimentalize the one-shot learning framework based on the Siamese network for the recognition of pneumonia. The study in [12] designs a lightweight Web-based platform that can run on a low-end server infrastructure. The platform consists of a screening application, a data collection module, a machine learning module, and a user notification module. The filter application process is designed for smartphone applications and Web pages. This can meet the need for simultaneous screening among different people. The data collection module is also available to a large number of simultaneous users. The machine learning module is used to answer questions about the risk of possible infection. The user notification module is applied to send specific messages on demand to inform the user of the outcome. For those with COVID-19 symptoms, the notification module will inform them of morbid progression. However, in case of malicious attacks on the platform and theft of user information, how to prevent the abuse of the platform remains an urgent problem to be solved.

Besides, wearable device technology can fulfill the task of remote screening. For example, the soft wearable device proposed by Lonini et al. [4], which is composed of a safer, softer, and reusable wearable sensor, records data by measuring the tiny vibration generated by heartbeat and breathing. Cardiac and respiratory characteristics are calculated to detect physiological changes induced by COVID-19.  Table 2 summarizes the remote screening methods or examples mentioned in this paper.

## 4. Intelligent Diagnosis of COVID-19

It is well known that X-ray and CT scans are the two standard methods for diagnosing COVID-19 by imaging. However, in the context of the outbreak of novel coronavirus, the use of CT scans and X-rays may pose risks to suspected patients and doctors, due to cross-infection. On the other hand, only reading a large number of image scans and manually drawing the outline of lung lesions will delay COVID-19 diagnosis [13]. Therefore, it is of great significance to develop an intelligent diagnostic system based on the IoT system, which can assist front-line doctors in jointly fighting against COVID-19.

**Figure 3:**
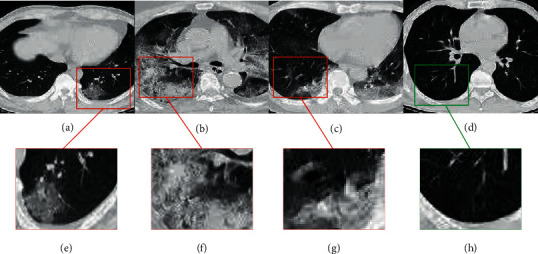
Chest CT images of laboratory-confirmed COVID-19 patients and 1 healthy subject. Patients' GGOs are highlighted with red borders, and normal subjects' GGOs are highlighted with green borders. (a–c) Lung CT images of COVID-19 patients in the early, advanced, and absorptive stages, respectively; (e–g) the parts of GGO in these three stages, respectively; (d) a CT scan of a normal lung; (h) a local magnification of a normal lung.

**Figure 4:**
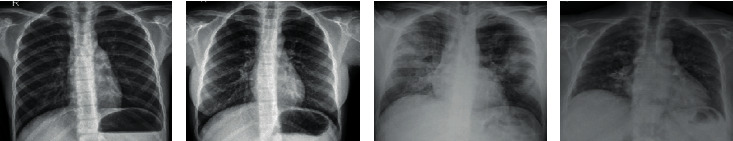
(a, b) The CXR image of normal people. (c, d) The CXR image of COVID-19 patients [16, 17].

Common chest CT findings of COVID-19 patients include ground glass opacity [14], consolidation, and pleural effusion.  Figure 3 shows CT images of patients and normal subjects with COVID-19 in different stages. According to the contrast, the CT manifestations of the lungs are often patchy or diffuse ground glass shadows in the early stage. In the progressive stage, the two lung diseases progress rapidly, multiple lesions fuse into large sheet consolidation, and the lesion density increases [15]. In the absorptive stage, the lesion range is slightly reduced, the density is reduced, and the fibrous cord shadow is visible. X-ray images of patients with COVID-19 are typically characterized by large and blurred lungs, as shown in Figure 4. It may be accompanied by the thickening of the cleft system at night with a small amount of pleural effusion. When the disease becomes more severe, diffuse consolidation shadows can be seen in both lungs, with white lungs and pleural effusion.

The premise of intelligent diagnosis by X-ray and CT scanning is image segmentation. The goal of segmentation is to separate the area or object of interest from the rest of the body for fixed-point measurement [18]. The study in [19] develops a dual-branch combinatorial network (DCN) for combinatorial segmentation and classification. Ranjbarzadeh et al. [20] propose a dual-path convolutional neural network for detecting and classifying COVID-19 infections by extracting global and local features. The study in [21] uses the Deep Network for Pulmonary Infection Segmentation (Inf-Net) to automatically segment the infected tissues in sections. In paper [22], they propose a lightweight CNN (Anam-Net) based on deformation deep embedding for the segmentation of anomalies in COVID-19 chest CT images. All of these methods can help to automatically segment the scanned image. As lung segmentation technology becomes increasingly mature, intelligent COVID-19 diagnostic methods will become even more reliable.  Table 3 lists some examples of intelligent diagnostic methods.

**Figure 5:**
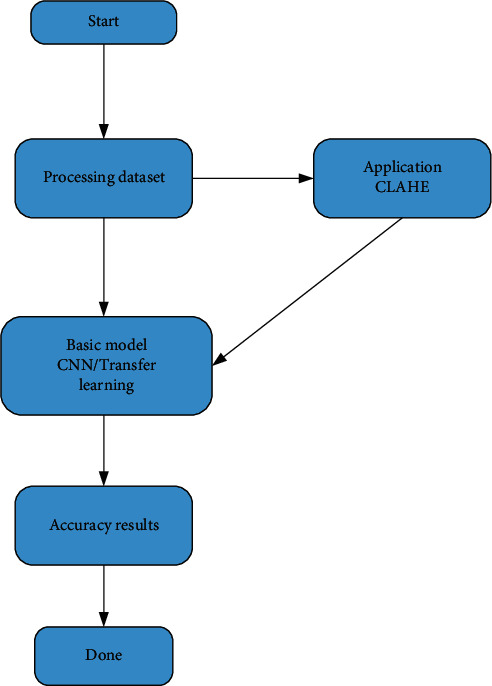
Distribution process of the combination of CNN and CLAHE.

**Figure 6:**
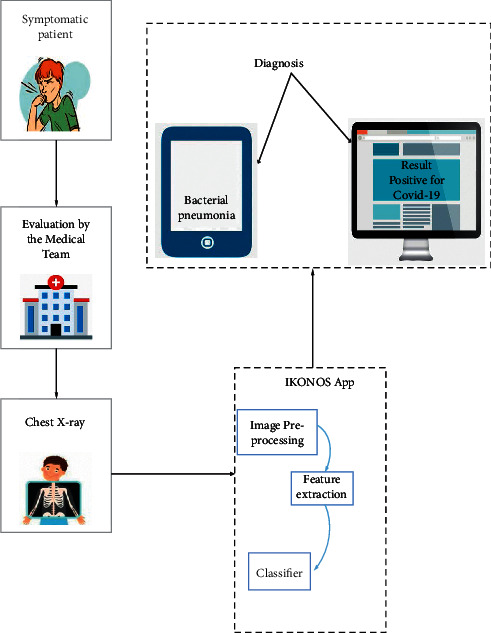
Chest X-rays of symptomatic patients can be loaded with IKONOS. The application consists of an intelligent system capable of extracting features and classifying images. The results can be viewed on the computer on which the software is installed.

**Figure 7:**
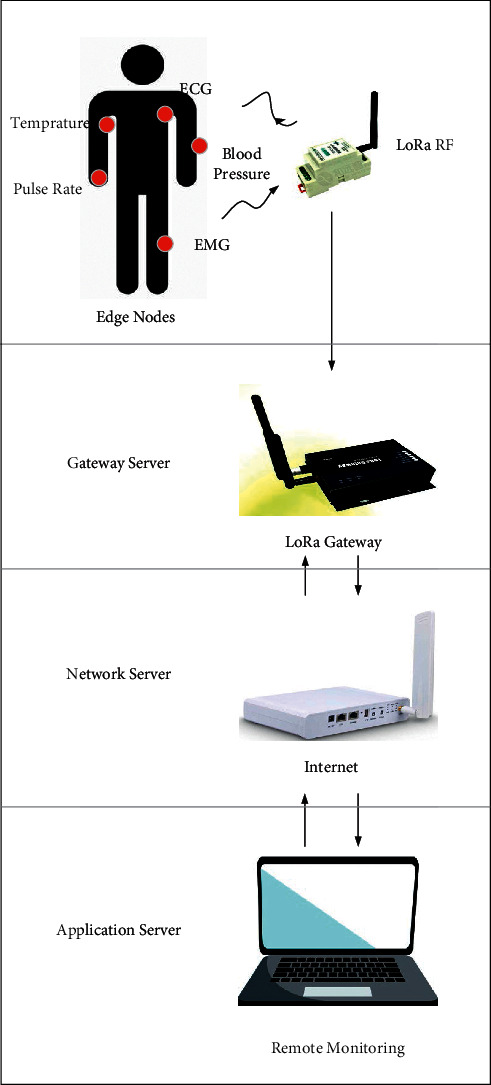
LoRa technology architecture.

Tang et al. [23] use neural network and digital image processing technology to design a lightweight classification model based on intersection attention mechanism, which integrates early screening, lesion assessment, lesion segmentation, and histogram of pixel distribution of lung and lesion. It takes only 0.4 seconds for each person to be diagnosed on average. Jiang's team [24] trains a VGG-16 convolutional neural network migration to build an intelligent COVID-19 diagnostic model, which is based on a small sample data set, and then uses CT scan images to distinguish between the early, late, and severe stages of COVID-19 in patients. However, Umri et al. [25] state that CNN has more significant advantages compared with VGG-16, and they propose to combine CNN and CLAHE to detect novel coronavirus in X-ray images. The advantage of CNN is that chest examination with novel coronavirus pneumonia combined with CLAHE can be performed by stages, as shown in Figure 5. Contrast-constrained adaptive histogram equalization and convolutional neural network are used to analyze the data sets. Gomes et al. [26] propose an intelligent system to support X-ray scan image diagnosis and develop IKONOS (a desktop application) to diagnose COVID-19 via X-ray image, as shown in Figure 6. After receiving an image, the doctor uploads it to the app, which uses texture and shape descriptors or classical classifiers for feature extraction and makes analysis by the intelligent system to identify COVID-19. Similarly, Narin [27] uses the ResNet-50 model of convolutional neural network (CNN) to carry out diagnostic research. With the help of the supervised learning method based on statistical learning theory (SVM algorithm), features can be also directly extracted to determine whether the disease is present [32]. The sensitivity of their experiment is higher than that of the study in [26] and thus makes it easier for doctors to reduce the rate of missed tests. The study in [33] proposes a new PSSPNN model for the diagnosis of COVID-19 patients. The result of the algorithm reaches 95.79%, which illustrates that the model can be used for early diagnosis. Singh and Singh [28] put an improved deep convolutional neural network for automatic diagnosis of COVID-19. The advantages which are brought by the combination of Wavelet Transform and Deep Network can be widely used to diagnose COVID-19 from chest X-ray images. The performance of this method is better than that of the commonly used methods, so it can be used for the effective diagnosis of COVID-19 disease [34]. In paper [29], they demonstrate that an iteratively built set of deep learning models can be used to intelligently diagnose COVID-19 via chest X-rays. Their combined use reduces the complexity of the model and the variance of the prediction values, thus promoting the adoption of the digital chest radiograph for the detection of COVID-19. The study in [30] introduces the trained deep learning model at the beginning, then designs two models (ResNet-50 and VGG-16) to extend CNN. Although there are many mistakes in the actual prediction, this study still provides a new research orientation of intelligent diagnosis. Wang et al. [31] propose a model based on CT image scanning for diagnosing COVID-19. Specifically, it is a module of prioritized attention residual learning (PARL) that trains the 3D-ResNet branch as a binary classifier for lung images. Its greatest advantage is that it can highlight the lesion area in the lung. With this advantage, this framework model can not only be widely used in the early intelligent diagnosis of COVID-19 but also be applied to other computer-aided detection, such as glaucoma and skin lesions in retinal fundus images. The intelligent assisted diagnosis model proposed [24] can quickly provide doctors with reference information and improve efficiency during the pandemic prevention and control period with its extremely high sensitivity and reliability in small data based on learning transfer technology. From another point of view, this method still has the problem of insufficient sample size and needs to be expanded. Bilandi et al. [35] put an intelligent, energy-efficient WBAN model for the diagnosis and monitoring of COVID-19 patients and another designed to classify COVID-19 patients with common cold. The proposed LoRa technology architecture is drawn in Figure 7. If the user is a COVID-19 patient, the WBAN will be installed on the user's body for continuous monitoring. On the other hand, LoRa modules act as relay nodes to improve the power efficiency and the network life of WBAN. Although LoRa has not yet been used on a large scale in China, it remains a market-driven technology choice as an effective packet transmission medium. In the future, LoRa will also have a large number of practical industrial applications in vertical fields such as intelligent cities and intelligent parks.

**Figure 8:**
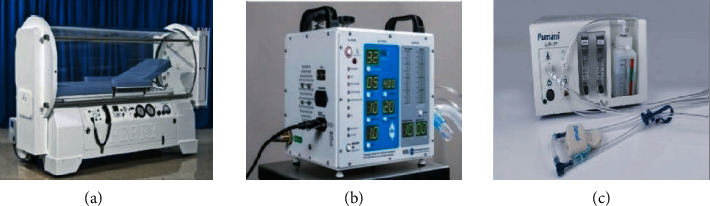
(a) Oxygen therapy devices; (b) ventilators; (c) CPAP.

**Figure 9:**
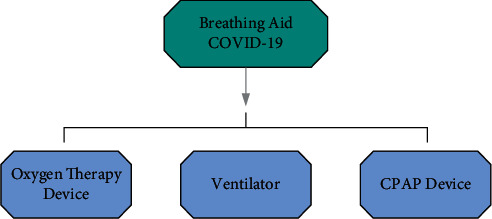
Breathing assistant available for COVID-19-infected patients.

During intelligent diagnosis, COVID-19 patients often have difficulty breathing, which is caused by hypoxemia. Severe dyspnea needs to be treated immediately with oxygen; otherwise, patients may suffer from cyanosis, a condition in which lack of blood supply can cause damage to various organs throughout the body. Severe patients may also have acute respiratory distress, respiratory failure, and other symptoms. Therefore, emergency rooms and other important medical facilities must be equipped with the necessary respiratory aids. Islam et al. [36] describe the latest respiratory aids, such as oxygen therapy devices, ventilators, and CPAP, as shown in Figure 8. For easy understanding, Figure 9 shows possible classifications. Oxygen in therapy apparatus is necessary for human body. When a person feels short of breath with an oxygen level lower than normal, he/she needs respiratory aids. Only timely use of respiratory aids can help healthcare workers to better diagnose and treat COVID-19 patients and save more lives. However, the existing respiratory aids have many deficiencies. For example, if they are used for a long time, a lot of water mists will appear on the inner wall of the oxygen mask, which will affect the medical judgment of the patient by the medical staff. Meanwhile, if the water mists accumulate to a water droplet, the water droplet left on the patient's face will cause discomfort. To overcome these deficiencies, Nanjing Yu Ru Meng Information Technology Co., Ltd., China, [37] has invented respiratory aid equipment based on AIoT, which includes a main body and an intake pipe. The intake pipe is set on the main body, which is equipped with a defogging and detection mechanism. The function of removing water mists on the inner wall of the main body is realized by using the defogging mechanism. The biggest advantage of the device is that it can detect in real time if the trachea has stopped delivering oxygen and can alert doctors at the same time. Not only can it monitor the user's breathing state, but it can also show strong practicability. It is believed that AIoT will be more and more widely used in intelligent medical treatment in the future.

## 5. Remote Intensive Care for Patients with COVID-19

**Figure 10:**
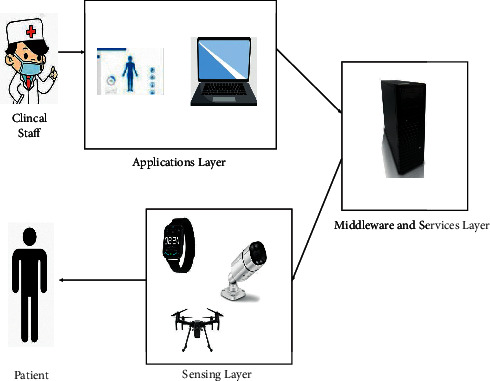
Examples of sensors and applications.

COVID-19 is dangerous mainly because of its wide spread and difficulty in treatment, so remote intensive care of patients is an excellent solution, which can help to avoid the unnecessary contact between the medical staff and patients. Accordingly, how can we achieve the full range of remote intensive care? Some researchers take the lead in proposing an AI recognition system based on the IoT system. Face detection algorithm is used to automatically detect and recognize faces. When detecting a COVID-19 patient, it will automatically search for facial and personal information to ascertain whether the information is stored in the database and will carry out remote monitoring continuously. The algorithm can help to obtain features through face recognition and real-time remote monitoring, which makes up for the deficiency of traditional monitoring system. The study in [38] proposes a system of detection and verification that uses deep learning (CNN) technology to recognize faces. The system uses the DPSSD face detector to perform the detection function and uses the integrated CNN to perform the positioning function. Afterward, Moorthy et al. [39] propose to use face detection algorithm for remote monitoring and tracking. Therefore, it is still difficult to use face recognition system to monitor patients remotely, because of great differences between faces, such as expression, posture, skin color, and position. There are great similarities between faces among such people as twins. In addition, during COVID-19, with the mask shielding, the accuracy and precision of the face recognition system for monitoring critical patients are greatly reduced. As to whether the face monitoring system can cover all the activity areas of patients, there are still some obstacles for developing countries or backward regions. By integrating wearable and unobtrusive sensors, the study in [40] extends the AIoT-based healthcare platform for remote intensive care of patients. The main work of the platform is to collect and process patient data to facilitate rapid clinical intervention. They conducted experiments using the framework PAR and found out that the platform could be used flexibly and continuously for remote intensive care. Moorthy et al. [39] built an AIoT-based system of intelligent devices and sensors for remote intensive care, which is able to track a large number of diseases and conditions. Although the above methods can effectively help to carry out remote monitoring, it remains to be verified whether wrong or missed diagnosis would occur if it is only based on the algorithm or face recognition system monitoring. Other sensors and application examples are shown in Figure 10.

**Figure 11:**
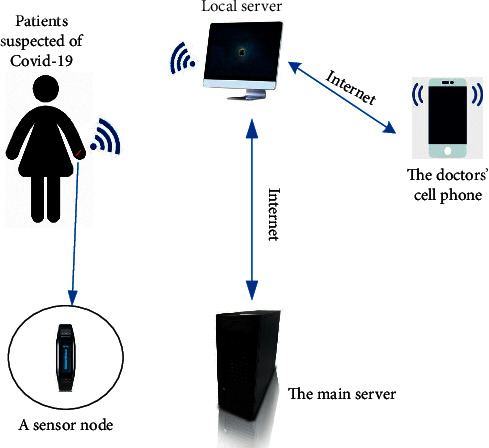
Remote intensive care architecture using a wearable sensor network system.

**Figure 12:**
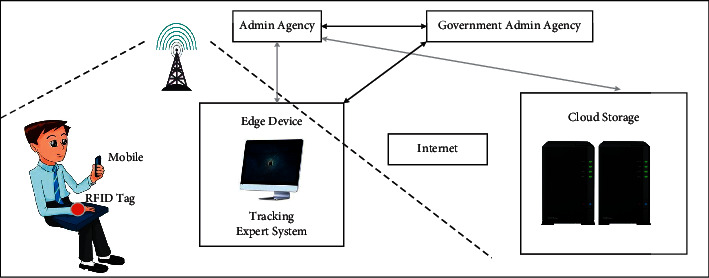
Structure of radio frequency identification model.

Remote intensive care for patients with coronavirus essentially embodies the development of telemedicine system [41, 42]. In fact, this way can not only help to solve the problem of insufficient critical care staff but also reduce the case fatality rate in ICU and the cost and waste of healthcare. Remote intensive care patients, according to telemedicine, can rely on wearable sensor devices to track vital signs and make “preliminary classification” based on the preliminarily collected data. That is, the data of the identified symptoms are sent to the doctor, so that the doctor can make the next diagnosis. Touil et al. [43] propose the use of a wearable sensor network system for remote intensive care of patients.  Figure 11 reveals the use structure of the system. LabVIEW software has developed an application for remote monitoring, in which a sensor node measures a patient's temperature and sends the information via Wi-Fi or Bluetooth to a local server for data processing. If the temperature is abnormal, a notice is sent to the doctor. In this system, the clinician is the master server, and all patients receiving remote intensive care are subservers. In this system, the side of the clinician is the master server, and the sides of patients receiving remote intensive care are subservers. Through the transmission of information between the subservers and the master server, the doctors are informed of the changes of patients' vital signs. That is because both diagnosis and monitoring take place on a doctor's personal mobile device, thus avoiding indirect transmission and playing a huge role in medical remote monitoring of suspected cases. Although remote intensive care for patients is a great promise, for such reasons as government policy, its development is still restricted. The study in [44] points out that in order to control the spread of the virus during the pandemic, the US federal government has launched necessary policy and regulatory reforms, which has led to the increasing use of telemedicine to provide patient care. Before the COVID-19 outbreak, telemedicine had been progressing slowly. The study in [45] puts forward the concept of telemetry system in a new way. The system can transmit data over the network without human-computer interaction. Pulse blood sampling kits proposed by paper [46] are also a means of remote screening. With the help of IoT technology, remote physical intensive care of COVID-19 patients can be performed via intelligent mobile devices. Rajasekar [47] exploits a COVID-19 case tracking model, based on the use of IoT and radio frequency identification technology. The model structure is shown in Figure 12. RFID tags or personal mobile devices are used to identify potential contacts.

All in all, with the novel coronary pneumonia spreading around the world, in order to provide remote intensive care, we must check the accuracy of face recognition system for remote intensive care before using the system. Facial recognition systems would also be more suitable for catching criminals or tracking missing people, which would reduce the workload of the police. Furthermore, remote intensive care applying AIoT-based sensors or applying the principles of telemedicine technology is entirely feasible. These technologies will play an increasingly important role in healthcare services even after the pandemic is over. They will still rely on remote operation, automation in the industry, manufacturing, AI industry, and other industries for wide applications.

## 6. AIoT and Social Life

### 6.1. Public and Social Management under the Pandemic

In the case of the outbreak of natural pandemic, the food supply chain has been hit hard because of panic buying, change of food purchase mode, traffic control, and so on. Therefore, more and more people pay attention to how to guarantee the safety of multilevel food supply chain. Problems in the Agri-Food Supply Chain (AFSC) are attributed to factory shutdowns and production reductions. This phenomenon has led to longer production and processing time and shortage of retailers, distribution centers, and transportation facilities. The study in [48] aims to model AIoT food safety and utilize intelligent AIoT technology for AFSC management. Meanwhile, the paper emphasizes that AFSC process tracking is very necessary. It provides such background information as the picking and processing time of agricultural products and the basic concept of the whole supply chain management process, so that people in the pandemic period can buy the food for eating without any worry. The study in [49] adopts three decision-based AFSC models, aiming to establish an AIoT-driven multilevel system based on ISM to deal with food safety risks. It can help to develop an IoT-driven food safety system. Zavala-Alcívar et al. [50] put forward the concept of “resilient strategy.” They believe that appropriate resilient strategy should be established to deal with events during the pandemic. The paper points out that AFSC must be readjusted to regulate production distribution and change the flow of workers. Only in this way can we improve the rapid delivery of agricultural products and shorten the cycle time. However, the supply chain of agricultural products is always inseparable from the labor force. How to formulate strategies to promote the development and dynamic capabilities of workers is always an issue worthy of attention.

In the mean time, the security of cold chain logistics has been taken seriously. In China, COVID-19 virus is frequently detected in imported cold chain food. People even suspect that COVID-19 is spread through imported seafood in the South Seafood Market in Wuhan. Under such impact, what should the cold chain industry do to develop itself? Yang and Zhang [51] make analysis in seven aspects: the physical level of supervision, logistics management, standardization system construction, insurance device, quality and safety of cold chain products, physical costs of cold chain, and construction of the snubbing information system. They believe that quality and safety of products are the primary factors that hinder the development of cold chain logistics. To this end, relevant departments should formulate logistics management regulations based on AIoT technology and use AIoT tracking and positioning functions to achieve effective monitoring of all links. During the pandemic, the inspection of transportation routes must not be omitted, and the safety of cold chain food must be ensured. On the other hand, countries need to pay constant attention to the training of cold chain logistics management personnel and establish a logistics standardization system. Ping and Na [52] propose the basic countermeasures to build China's logistics supply chain system, strengthen strategic cooperation between upstream and downstream enterprises in the supply chain, optimize cargo management policies, and improve import and export efficiency. It is worth noting that we pay much more attention to development than to safety.

**Figure 13:**
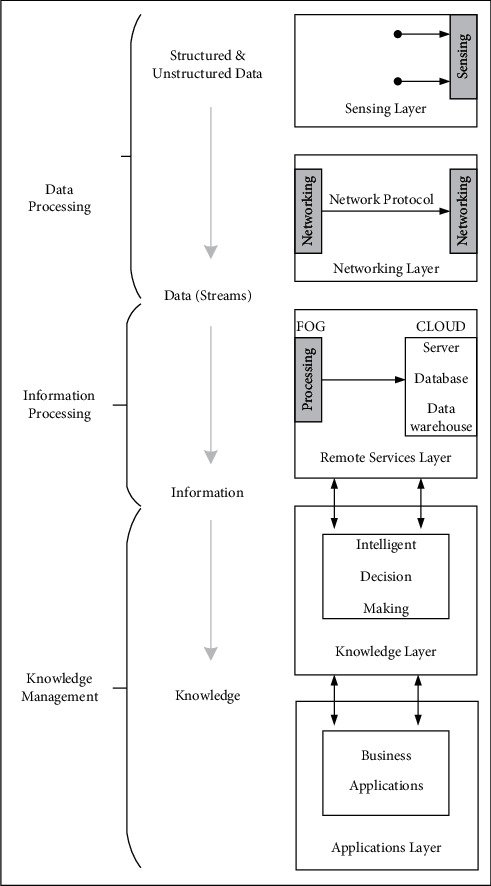
Five-tier architecture of the Internet of Things.

Even in a pandemic situation, it is invariably important to maintain the stability of the public society and promote the continued development of the public society. Therefore, it is very necessary to exploit AIoT to improve the intelligence of infrastructure and maintain social security and stability. The study in [53] proposes the construction of intelligent infrastructure. For example, it can be used in buildings, apartments, hotels, large shopping malls, and so on. The advantage of intelligent infrastructure is that it can inform the administrator by measuring body temperature through infrared scanning, as well as informing the administrator of more details through facial recognition. On the other hand, intelligent infrastructure needs to be equipped with advanced equipment, such as Swann Thermal Sensing PIR Security Camera, Swann 1080p Alert Indoor Security Camera, Yobekan KV-11 Non-Contact Infrared Thermometer, Fluke 568 Contact and Infrared Temp Gun, and PerfectPrime IR0001 Thermal Camera. Suppose that such a building can be built to ensure the safety of people's homes in a pandemic era. Uslu et al. [54] believe that it is necessary to establish the technical infrastructure and suitable environment for the development of an intelligent hospital. Intelligent hospital takes advantage of AIoT, data analysis, and key technologies of personalized services to achieve self-management ability. The current AIoT architecture uses a five-layer model, as shown in Figure 13. The architecture of an intelligent hospital consists of a perception layer, a network layer, a remote server layer, a knowledge layer, and an application layer. Compared with the traditional three-tier structure system, the five-tier model makes up for the disadvantages of high energy consumption and low communication capabilities.

**Figure 14:**
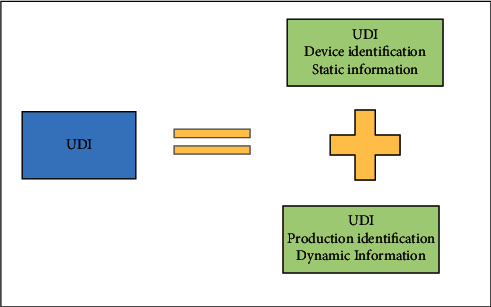
Composition of UDI.

**Figure 15:**
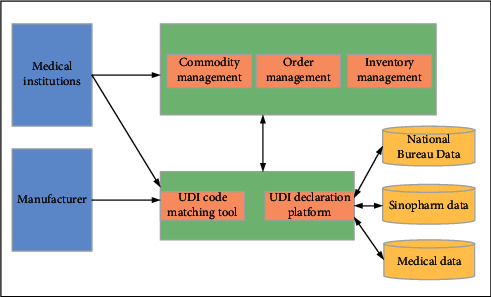
Design plan of the specific UDI platform.

The study in [55] proposes the scanning technology of unique medical device identification (UDI). UDI is the first “special medical device identification system” established by the US FDA. Its composition is shown in Figure 14. They hope to use UDI to build a new model of medical consumables management based on AIoT. Its construction requires three parts, namely, establishing a basic database, understanding the material procurement process, and implementing material supervision. In this way, it can easily realize refined management of consumables and control the construction cost of intelligent hospitals. Wang [56] effectively integrate the data of the State Food and Drug Administration for manufacturing and operating companies and medical institutions. In order to create a big data platform, Figure 15 shows the specific UDI platform design plan. This program can help to realize the comprehensive lean management of consumables in the hospital and all-round tracking of consumables outside the hospital. The paper also shows the implementation of the AIoT management mode with all consumables being traceable. Clinical departments can have clear inventory records and usage records for their own secondary libraries. Suppliers can inquire about the inventory of their own products in the hospital and check for omissions in time. Take the West China Hospital of Sichuan University in China as an example. They are trying to establish a warehouse system with detailed division of labor, build a shared management platform, and create an intelligent logistics chain based on UDI. These new attempts have reduced operating costs and provided safety guarantees for the hospital. On the other hand, there are many advantages in using UDI to help hospitals. First of all, information about the storage of medical consumables and the use of each department on the system platform can be clearly seen. During COVID-19 pneumonia, doctors do not need to manually register, but automatically provide data information to hospitals, thus saving labor costs. Second, the system gives an intelligent reminder of products' validity period, which ensures the safety of medical consumables in the special times. Finally, the information about the use of consumables forms a closed loop from the procurement warehousing to information scanning and registration, from the electronic record system to the scanning code billing when patients use it. It is beneficial to the improvement of the utilization rate of medical consumables. These advantages can help hospitals to actively fight against COVID-19 pneumonia. It is believed that in the future more intelligent hospitals will use the UDI management model to become more scientific, reasonable, and standardized.

**Figure 16:**
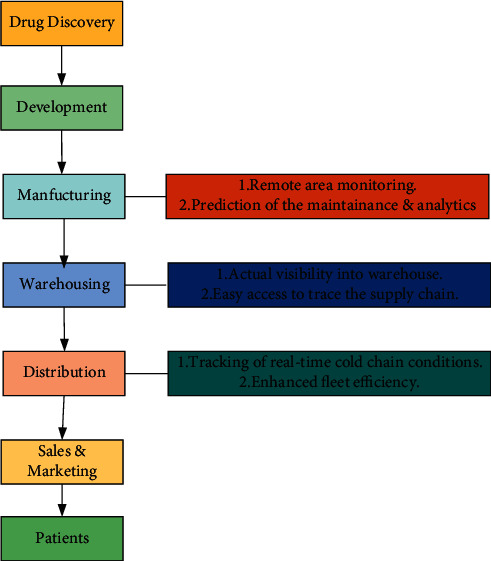
Application of AIoT in different pharmaceutical processes.

Intelligent hospital needs the support of the pharmaceutical industry, but with the massive use of pharmaceutical products and medical equipment, it has brought tremendous pressure to the pharmaceutical industry. Therefore, more and more researchers are exploring the application of AIoT in the manufacturing of drugs. They hope to utilize the AIoT to help the medical industry improve production efficiency to reduce production costs. The study in [57] points out that the biggest advantage of using the intelligent AIoT is that it can use cloud tracking technology to understand patients' compliance with prescribed drugs, thus narrowing the gap between drug suppliers and consumers.  Figure 16 shows the application of AIoT in different pharmaceutical processes. As an important key point of the pharmaceutical industry, warehouses are usually distributed throughout the country to ensure the continuous and timely flow of drugs. However, it is a very difficult task to accurately track the products in the warehouse and know their transportation routes, such as inventory of products and product warehousing. Nevertheless, if AIoT is used together with radio frequency identification technology and wireless video monitoring system, the problems can be easily solved, and the intelligent and economical warehouse management can be realized. Most importantly, the application of AIoT to the pharmaceutical supply chain has great advantages in terms of preventing counterfeit drugs from flowing to the market. From 2D barcodes and RFID tags used in pharmaceutical production to intelligent packaging used in pharmaceutical retail, AIoT technology can be utilized to prevent the circulation of counterfeit drugs in the consumer market.

In fact, many countries and regions have taken actions and achieved good results. Radanliev et al. [58] point out that the application of COVID Symptomatic Trackers in the UK has reached 700,000 application downloads and registrations within 24 hours. This demonstrates the precise and timely surveillance of COVID-19 in the UK and also demonstrates that the speed and scale of AIoT have improved the NHS's ability to monitor high-risk patients. In China, the “health code” hidden in Alipay is even more popular. Before using common transportation means, passengers are required to show the color of their code to the staff. Green code means health. Red or yellow code means the code holders are from areas with high and medium risks and need to be self-isolated or be placed under supervision. The code determines the whereabouts of the past 14 days based on the location history. However, whether such an approach raises privacy and security issues in digital pandemic control remains to be tested. South Korea, as a country of early outbreak, has deployed a tracking app called “CO100,” which notifies people of known cases within 100 meters of their location. By observing the measures that these countries are using to respond to the pandemic, we can easily find out that AIoT technology plays a great role in it, helping each country to maintain social stability and manage public affairs.

### 6.2. The Role of AIoT in Resumption of Work

**Figure 17:**
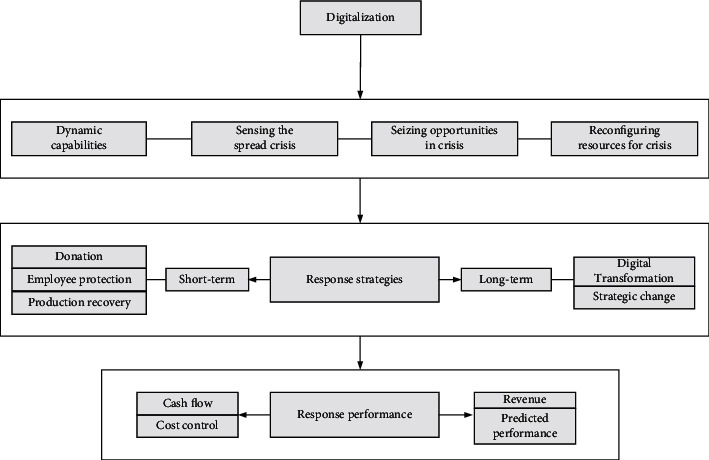
Theoretical framework of SME digitization and crisis response.

As the pandemic is slowly brought under control, regardless of the size, businesses are starting to return to work. Then, how can businesses recover and develop in the new era of the pandemic? Guo's team [59] raises the theoretical framework of SME digitization and crisis response, as drawn in Figure 17, and proposes three approaches for future research. First of all, because of the lockdown of cities by governments, the long-term shutdown of enterprises' production capacity, the threat posed by the pandemic to the service industry, and the sustainable damage caused by the pandemic, all enterprises are required to have certain ability in ideological construction and crisis public relations. Second, it is necessary to improve the digitalization degree and business model of small- and medium-sized enterprises. For example, working from home can avoid the expense of renting work space and even decrease the contact between people as well. In the meantime, this is very unfriendly to the kind of cooperation-intensive business. Compared with microenterprises, multinational corporations bear more pressure and risks. The study in [60] believes that MNCs must wake up from COVID-19 for a booming economy. They propose that MNCs should grasp this opportunity, make good use of AIoT, reform their internal social responsibility functions, take sustainable development as the goal, and establish new alliances.

In addition to ordinary businesses, COVID-19 has brought huge losses to special businesses such as tourism and catering. Because of COVID-19, the tourism industry has come to a standstill, with its economic performance having collapsed. Therefore, measures must be taken to save the current situation. The study in [61] puts forward several suggestions. First, the government should play a leading role in guiding and encouraging the tourism industry and departments at all levels to build confidence. Then, relevant policies should be launched and implemented to promote the development of intelligent tourism, so as to combine tourism and AIoT perfectly, which can be successful. This transformation can be achieved by using new media. The study in [62] emphasizes the application of new technologies in the tourism industry. They point out that many tourism enterprises use online activities to recover losses, such as online theme planning, cloud tourism, and cloud live broadcasting. Many traditional tourism companies are transitioning to online travel ones (OTAs). As the organic combination of tourism and AIoT, these changes promote the change of concept, accelerate the digital integration process of traditional tourism industry, and facilitate the high-quality development of China's tourism industry. Take China for example, Ctrip launched a blind box ticket, where participants book tickets on the app and the system randomly selects the destination for a short trip. This encourages people to travel to some extent when the outbreak of COVID-19 is under control. As for the catering industry, to prevent the spread of the virus, people are not allowed to eat in the restaurants, and due to restrictions on travel the number of customers has decreased. Therefore, it is necessary for the catering industry to be transformed. In paper [63], enterprises are expected to build an AIoT-based business model, so that customers can adopt online restaurants and order food by scanning the code online. They should vigorously ensure the safety of takeaway food and carry out “noncontact takeaway.” At the same time, in view of the changes brought by the pandemic, catering enterprises should avoid sensitive food ingredients and wild animals. In general, it is difficult to avoid the impact of the pandemic, but each enterprise can take actions to stand the test after the outbreak and minimize the loss. COVID-19 will bring about a reshuffle in industries: those companies that are unable to seek breakthroughs in time will be eliminated, and only those companies that are brave enough to innovate and change will survive.

## 7. Using AIoT-Based Technologies against COVID-19

### 7.1. 5G Technology for New AIoT Applications

All over the world, the number of people influenced by COVID-19 increases rapidly, so immediate actions need to be taken to control the devastating result of the COVID-19 outbreak. The researchers explore the use of 5G, robotics, and other technologies that they believe can help to mitigate the adverse influences of pandemic and speed up the recovery of society.

**Figure 18:**
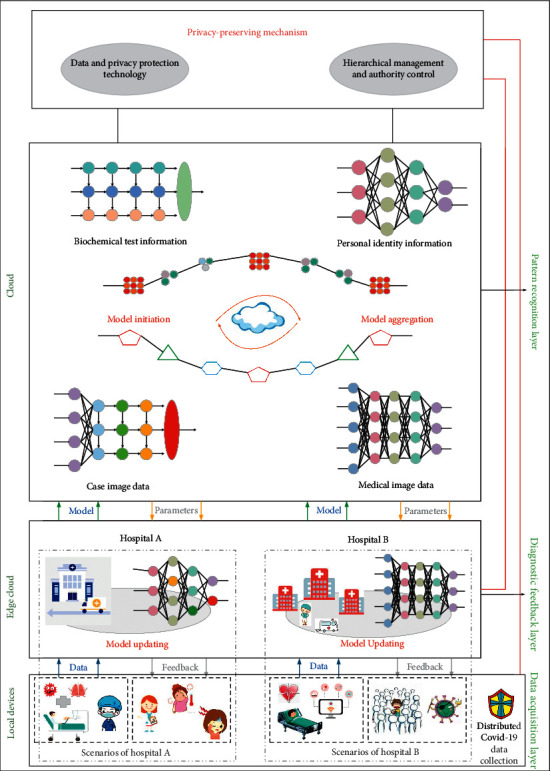
Architecture of auxiliary diagnosis based on federated learning.

**Figure 19:**
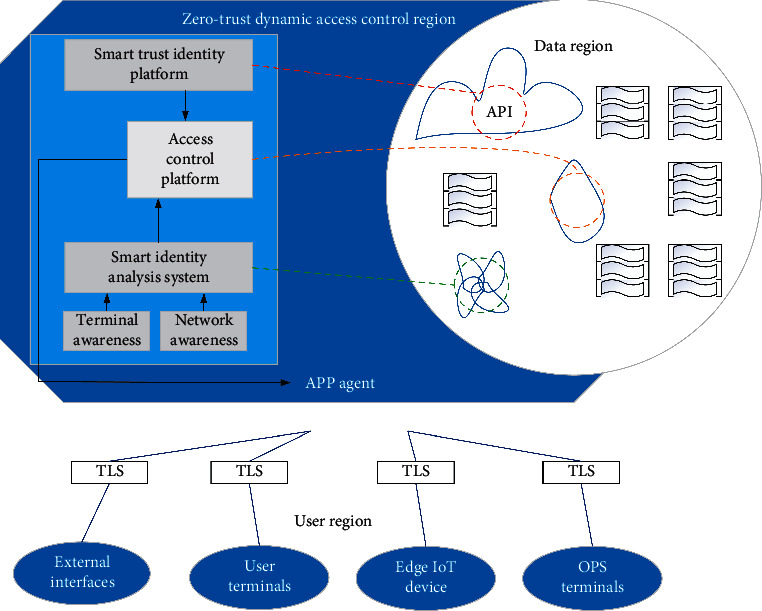
Basic zero-trust security awareness and protection system architecture.

As the fifth-generation mobile communication network, 5G has super extensive applications in the current situation, with characteristics of low-power consumption and high speed. The IoT connects physical objects to the network and finally realizes intelligence and automation, thus freeing a large number of labor forces. These two popular technologies are also closely related: 5G will accelerate the pace of entering the IoT era. Once limited by the application of traditional mobile communication, IoT can now be implemented under 5G. In turn, IoT will be the main driver of 5G development. Accordingly, how will 5G work today in the times of COVID-19? The study in [64] hopes to adopt 5G technology to assist the diagnosis of COVID-19 and proposes an architecture of auxiliary diagnosis, which relies on 5G-enabled federated learning for multiple institutions and central cloud collaboration. As shown in Figure 18, the architecture mainly consists of three parts: data acquisition layer, diagnostic feedback layer, and pattern recognition layer. The data acquisition layer uses 5G technology to automatically collect and transmit data for the local devices deployed in hospitals. Then, these locally collected data are uploaded to servers at the edge nodes and are used for recognizing and diagnosing diseases. Finally, the pattern recognition layer generates a generic model by training node data for each hospital. Only after the three steps can auxiliary diagnosis be realized. This architecture is beneficial for node hospitals to realize low delay and high performances of disease diagnosis. The study in [65] proposes a protective system of safety awareness which adopts zero-trust architecture (ZTA) for a 5G-based intelligent medical platform. The system is composed of user region, zero-trust dynamic access control region, and data region. The architecture is drawn in Figure 19. In consideration of a huge number of AIoT connected devices, the system adopts the access technology of ZTA. By using edge computing, it realizes authentication and access control of the terminal, so as to detect and deal with the illegal or false connection in time.

**Figure 20:**
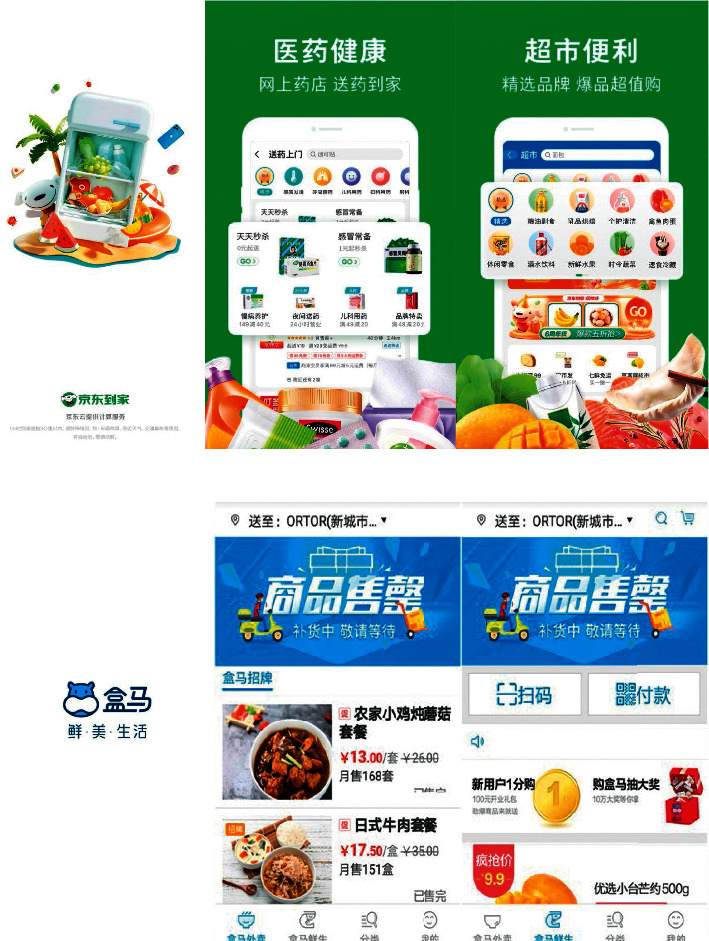
“JingDong Home” and “Hema Fresh” platforms.

**Figure 21:**
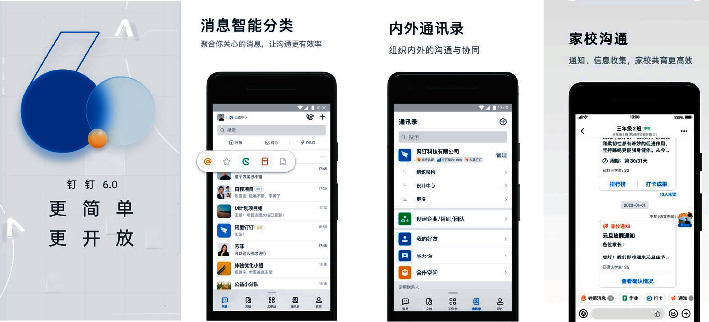
“DingDing” app.

5G provides a strong technical support for the development of intelligent medical applications. During the pandemic, 5G technology can aid the medical industry to achieve multidisciplinary consultation, intelligent diagnosis of medical images, remote monitoring, and other medical application scenes. However, for such reasons as the concentration of medical resources, highly intensive personnel, and complex information system, there are still many challenges for the application of 5G in the medical industry. In fact, not only can 5G technology help doctors to fight against COVID-19, but it can also drive lifestyle changes. During COVID-19, in order to avoid being exposed to viruses, new digital economies such as online food ordering, online entertainment, and online shopping have become active and given birth to new jobs and employment methods. Take China for example, COVID-19 has brought a development outlet to livestream e-commerce enterprises. Through livestream, the contactless transaction of online selling and offline receiving goods has come true. “Contactless” delivery platforms such as “JingDong Home” and “Hema Fresh” have also progressed rapidly (Figure 20). Meanwhile, 5G-based telecommuting is becoming more and more popular among employees. According to the Statistical Report on Internet Development in China released by China Internet Network Information Center (CNNIC), as of December 2020, the number of telecommuters in China reached 364 million due to the impact of COVID-19. Online work can reduce employees commuting time and cost and allow employees to have more free time. Enterprises also do not need to rent expensive office space and reduce their operating cost. In terms of education, 5G-based online education offers opportunities for students to learn without interruption. These platforms allow real-time interaction between students and teachers, replacing offline classes with high-quality videos. In this special time, more and more students in China have registered for software named “DingDing” (Figure 21), with which students can attend online classes simultaneously. Moreover, teachers can also assign homework and test practice to grasp the learning outcome of students. Notably, the application of 5G has positive influences in many aspects. However, potential challenges cannot be ignored, starting with security and privacy issues. Video records of telemedicine and shopping records of online food orders may contain personal addresses, and such sensitive information is extremely vulnerable to hackers. Secondly, the quick deployment of new applications, such as online education platforms and online e-commerce platforms, can easily cause network breakdown. Finally, the existing 5G technology is still in its infancy, and many poor countries have not deployed 5G. In the future, 5G technology will be developed and applied in more fields.

### 7.2. IoT Terminal Robots

Remarkably, it is found out that the application of intelligent robots in the medical field can reduce the burden of medical staff and decrease the risk of cross-infection. The combination of intelligent robotics and medical technology boasts a great application prospect, which is suitable for social progress.

#### 7.2.1. Health Care

**Figure 22:**
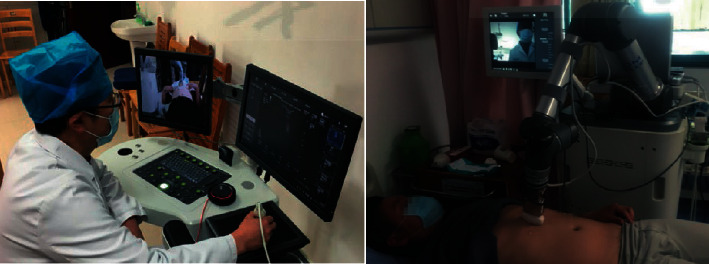
Remote ultrasonic inspection of 5G robots.

**Figure 23:**
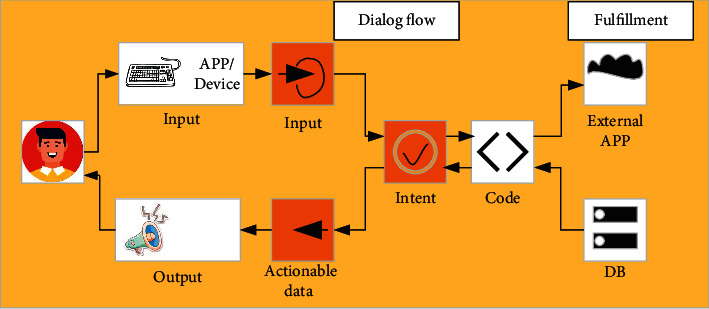
Specific workflow of COVID-19 screening in AHM.

In terms of remote diagnosis, the study in [66] proposes a low-cost microrobot that can be easily assembled and monitored remotely, aiming to replace medical staff with the robot to take throat swabs from suspected patients. About medical treatment, the study in [67] probes the clinical value of 5G-based robots (Figure 22). In this paper, they carry on a comparative experiment between the remote machine inspection and the conventional ultrasound examination. The results show that the telerobotic ultrasound examination takes more time, but the results are similar to those of traditional ultrasound. As a result, 5G remote ultrasound robots can carry out regular ultrasound examinations for quarantined patients. The study in [68] is working on intelligent automated health machines (AHM), which can offer all virtual health checks in contact with a doctor or specialist online. Due to restricted travel in quarantined areas, AHM can be installed to help residents to receive medical treatment when they cannot go to hospitals. Residents use a personal identification card to enter the AHM facility, and then chatbots ask residents about their symptoms. Meanwhile, it is time for the infrared sensor to work. If the temperature rises above the set point, the infrared sensor will initiate an online video telephone with a doctor automatically. When the case is serious, AHM will book a nucleic acid test or an ambulance service for the resident in time and send the results to the resident's mobile phone to ensure that he/she knows his/her own situation. On the premise of the patient's permission, his/her family members can also receive the message of his/her situation, so that they will not worry too much. After residents leave the AHM, the system will also activate UV disinfection to avoid unnecessary cross-infection. The specific workflow of COVID-19 screening in AHM is drawn in Figure 23. Systems like this can be deployed not only in isolated areas but also in public places such as communities, schools, shopping malls, and office buildings. With the use of AHM systems, millions of lives can be saved in this pandemic. Next, how to reduce the maintenance and repair cost of AHM system and how to reduce the misjudgment rate of AHM system are becoming the directions of future research. In another aspect, for patients with mild symptoms who are treated at home, the intelligent voice robot can help them to overcome difficulties. Telemedicine-based voice robots are acting as family doctors and caregivers, providing fundamental medical and lifestyle services to patients. In particular, the vocal robots use natural language to communicate with old patients, avoiding the loneliness of isolation. In fact, the application of vocal robots is quite reliable in detecting many diseases, so that old patients can be treated at home without causing any danger to doctors.

#### 7.2.2. Disinfection of Facilities

**Figure 24:**
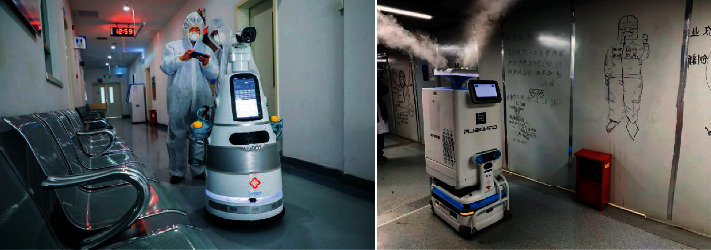
A sanitizing robot cleaning a hospital with ultraviolet light.

In terms of service and maintenance, robots can substitute professionals to complete a variety of daily tasks. For example, serving robots can keep track of drug inventories voluntarily and aid in placing medicines and medical equipment properly. Cleaning robots can take over simple tasks in hospitals where frequent disinfection is needed. This will decrease the exposure of healthcare workers to contaminated materials. In an ordinary hospital room, cleaning robots can kill all viruses and bacteria in a few minutes. The study in [67] emphasizes the sterilizing effect of intelligent disinfecting robots after surgeries. Compared with traditional manual disinfection, the intelligent disinfecting robots, which use ultraviolet light combined with hydrogen peroxide or hypochlorous acid, can effectively disinfect the air and the surface of environmental objects in the surgery room. The whole process of intelligent control is achieved to ensure the safety of personnel. Similarly, disinfection robots are also suitable for disinfection of other public places, such as hotels, shopping malls, and schools (Figure 24).

#### 7.2.3. Delivery of Food or Medicine

**Figure 25:**
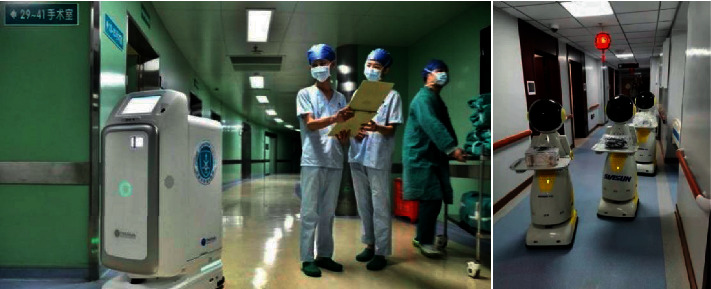
Robots delivering food and picking up medicine.

Besides, social robots also provide more and more help for hospitals. For example, robots can aid nurses in isolation wards with delivering meals and dispense medicine (Figure 25), which enhances work efficiency. Most importantly, when robots enter isolation wards, they do not need to wear thick protective clothing, thus saving economic and human costs. Robots dispense goods instead of medical workers, allowing workers to devote more energy to treating and caring for patients. In many hospitals, patients are still transferred in the traditional way, relying on medical staff or family members to being lifted to stretchers or wheel chairs. Not only does this approach require two or three people to complete, but it is also extremely likely to cause secondary injury to the patients. Intelligent robots can smoothly lift the elderly from the bed to the wheelchair or other places. It is helpful because it can reduce the manual labor of health workers, so that they can focus more of their energy on the recovery of patients. The study in [69] designs robots that can travel freely in and out of the hospital along the designed route. They can track and record the activity sites of suspected patients and help to transfer patients from ambulances to the wards without human intervention, thus reducing unnecessary labor. For example, the work in [70] studies the fully autonomous centralized multirobot composed of hexapod walking machine and six-wheeled mobile machine. In the method proposed by them, hexapod robots are used to scan and map the field hospital, draw the path, then deliver medical goods, and enter various places according to predefined maps and paths, and two different platforms are used to navigate robots at the same time.

**Figure 26:**
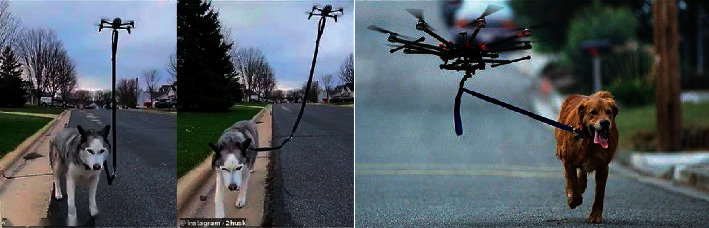
Robots helping to walk dogs.

**Figure 27:**
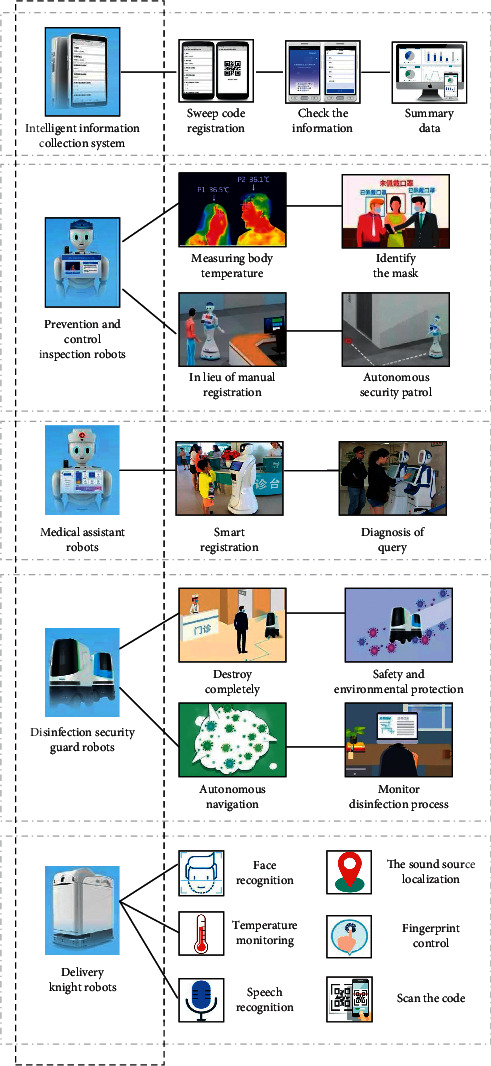
Neusoft Group's five invented “antipandemic” robots.

During the COVID-19 periods, intelligent robots are not just suitable for the medical industry. In many areas, people's travel is severely restricted in order to maintain social distance. For families with pets, there is the problem that they are not able to walk their dogs outside. In this case, using a robot to walk the dog is a good option (Figure 26). Robots can clearly do more meaningful things than walking dogs, such as delivering fresh fruits and vegetables to gated communities and acting as security guards to monitor the comings and goings of residents. Obviously, many enterprises in different countries are aware of the important role of robots in pandemic prevention. Take China for example, Neusoft Group has developed five types of “antipandemic” robots (Figure 27), which are the intelligent information collection system, prevention and control inspection robots, medical assistant robots, disinfection security guard robots, and delivery knight robots. The intelligent information collection system includes information filling, real-name checking, temperature measurement, and other functions, which can greatly help to control the number of people entering and leaving hospitals. The prevention and inspection robots are mainly used in the front-line positions of service places. They can authenticate the ID card with real name and accurately measure the body temperature of visitors as required. The robots have other functions. For example, they can set up fixed-point patrol routes to avoid human contact as far as possible. In the case of unattended service places, the first line of defense will be set up at any time. The medical assistant robots are oriented to the medical industry. In the absence of manpower, the robots distinguish fever patients from normal patients and enable healthcare workers to protect themselves while fighting against the pandemic. In addition to these, the robots also provide a series of operations including registration, payment, and inquiry. Disinfection security guard robots can monitor the workflow in real time and generate a complete work log to ensure efficient disinfection of indoor hygiene. After the current mission is completed, people can replan their work route. Delivery knight is a delivery robot used in medical settings, assisting doctors in accurately delivering drugs or meals to special wards, and reducing the risk of people being exposed to sources of infection. The advantages of delivery robots are that they can bear different weights of goods, and the boxes are in a closed state, without causing pollution. This means being 10 times more efficient than manual delivery, thus ensuring accurate and efficient delivery during the outbreak of the pandemic. It is believed that more and more countries and regions will apply robots to various fields in the future. Meanwhile, with the development of science and technology, intelligent robots will follow the needs of society.

## 8. Conclusion and Future

Although COVID-19 has wrought havoc to more than 30 countries and regions in the world, the good news is that the 21^st^ century is an advanced scientific era. Researchers are working to make new breakthroughs in screening, diagnosis, and monitoring of COVID-19. Intelligent technology based on IoT systems has proven to be an extremely valuable resource, with applications ranging from wearable technology to remote screening, from intelligent diagnosis to remote intensive care of COVID-19 patients. Wearable devices are convenient for measuring individuals' health conditions, and the data can be fed back to hospital units in a timely manner by means of positioning systems. Remote screening can avoid the mutual contact between medical staff and screening receivers, as well as improving the screening efficiency, so that patients can be early detected for prompt isolation. Intelligent diagnostics lightens the workload of front-line doctors by quickly identifying COVID-19 from X-ray and CT scanning, so that patients can be treated as soon as possible. Subsequently, AI image acquisition will enable patients to obtain high-quality images even at low radiation levels. Benefiting from the development of telemedicine, remote intensive care can solve the problem of timely treatment, as well as effectively reducing the waste of medical resources. It is also hoped that, in the future, remote systems will be used not only in medical care, but in all aspects of people's lives. In the latter part of the paper, AIoT and social management are mentioned. With the continuous development of AIoT, this technology can help public social management and enterprises to resume work and production in the event of the pandemic. Countries have been searching for strategies to return to what they were before the pandemic. Facts have also proven the feasibility of AIoT. In terms of public social management, it has been used to improve the logistics industry chain, build a new infrastructure, and help the pharmaceutical industry to resume its development. In terms of large, medium, and small enterprises, the theoretical framework has also been improved based on it. The paper also focuses on emerging technologies, such as 5G and robotics, which it believes will be effective in combating the pandemic by combining AIoT with medical technology.

In the future, more intelligent devices based on AIoT will emerge to fight against pandemics, for example, the use of cloud technology to achieve the integration of COVID-19 screening, diagnosis, monitoring, etc. and the use of integrated algorithms to predict the recurrence of the pandemic and take quick actions. It is more likely that a comprehensive infrastructure that integrates AIoT and cloud computing, which can jointly fight against diseases that pose a global threat, will be built.

## Figures and Tables

**Table 1:** Summary of the advantages and applications of wearable devices.

Authors	Advantages	Applications
Mohammed et al. [1]	It detects the coronavirus automatically from the thermal image.	Monitoring the screening process
Fyntanidou's team [2]	Vital signs can be continuously monitored.	Prioritizing emergency department patients
Ashraf et al. [3]	It warns individuals of their fever in advance.	Remote monitoring of patients
Lonini et al. [4]	The sensor can be placed on the sternum to better fit the body.	Screening high-risk individuals
Bassam et al. [6]	The health and recovery of COVID-19 patients can be tracked.	Predicting situations and raising the alarm
Dhadge and Tilekar [5]	The device can send alerts to prevent the virus from developing.	Predicting disease progression

**Table 2:** Methods and results of remote screening mentioned.

Authors	Methods	Results	Features
Schinköthe et al. [7]	The cockpit (C19CC)	Identifying telemedicine options, which can quickly improve patient care	Improving care and safety for people with COVID-19
Tan and Liu [8]	Facial recognition system using thermal imaging	Ability to identify and track patients	Helping to control the spread of COVID-19
Hou et al. [10]	Portable scanner + subspace multilayer networks	Achieving test data accuracy of more than 96%	Improving energy efficiency
Mulchandani et al. [11]	The one-shot learning framework based on the Siamese network	Diagnosing coronavirus from tonsillitis effectively for mass-screening	Used to detect susceptible patients at a very early stage
Chilipirea et al. [12]	Scalable COVID-19 screening platform	Ability to collect and analyze data for more than 200,000 people per minute	The main feature being the use of distributed, lightweight design
Lonini et al. [4]	Use of soft wearable devices	Rapid screening for COVID-19	Reducing the risk of pneumonia among healthcare workers

**Table 3:** Methods applied to intelligent image diagnosis of COVID-19 and the results of each method.

Authors	Database used	Obtained results	Highlights
Tang et al. [23]	247 COVID-19 patients and 152 other pneumonia patients	In the algorithm model, the average diagnosis time per person has been reduced to 0.4 s.	It has high application value.
Jiang and Xu [24]	CT images of patients diagnosed with COVID-19 in Zhongnan hospital	The sensitivity of the intelligence-assisted diagnosis model is 96%.	Comprehensive diagnosis accuracy is high.
Umri et al. [25]	GitHub and Kaggle website	The accuracy is 98%.	Compared with VGG-16, the effect of CNN is better and significant.
Gomes et al. [26]	Kaggle website	The average accuracy is 89.78%; the average sensitivity is 89.79%.	Computing costs are lower than those using deep learning techniques.
Narin [27]	Kaggle website	The highest sensitivity value is 96.35%.	It is beneficial to reduce the doctors' misdiagnosis rate.
Singh and Singh [28]	6,500 chest X-rays	The overall accuracy is 95.83%.	It is used to diagnose COVID-19 from chest X-ray images.
Sivaramakrishnan et al. [29]	CXR images of children aged 1 to 5 years collected at Guangzhou Medical Center	The highest accuracy is 99.01%.	Weighted average performance significantly improves performance.
Hernandez et al. [30]	https://www.sirm.org/category/senza-categoria/COVID-19/	The accuracy rate is about 90%.	It provides a completely new way of thinking.
Wang et al. [31]	Chest CT scans of 251 patients with corresponding voxel-grade lobes	The proposed method has an accuracy of 93.3%.	It detects the most accurate location of the lesion area.

## Data Availability

No data were used to support this study.
